# Opioid use disorder (OUD) and treatment for opioid problems among OUD symptom subtypes in individuals misusing opioids

**DOI:** 10.1016/j.dadr.2024.100220

**Published:** 2024-02-14

**Authors:** Emily A. Miller, Angela M. DeVeaugh-Geiss, Howard D. Chilcoat

**Affiliations:** a Virginia Commonwealth University School of Pharmacy, 410 N 12th St, Richmond, VA 23298, USA; b Indivior, Inc., 10710 Midlothian Turnpike, Suite 125, North Chesterfield, VA 23235, USA; c Johns Hopkins Bloomberg School of Public Health, 624 North Broadway, Baltimore, MD 21205, USA

**Keywords:** Latent class analysis, Opioid misuse, Opioid use disorder, Symptomatology, Treatment

## Abstract

**Background:**

In 2021, approximately 60 million individuals worldwide and 9 million individuals in the United States (US) reported opioid misuse. In the US, 2.5 million have OUD, of which only about a third receive any substance abuse treatment. OUD is often regarded as a monolithic disorder but different opioid problem subtypes may exist beyond DSM-IV/5 criteria. Understanding the characteristics of these subtypes could be useful for informing treatment and intervention strategies.

**Methods:**

Latent class analysis was used to identify OUD symptom subtypes among persons in the US who reported misusing prescription opioids or heroin in the 2015–2018 National Survey on Drug Use and Health (n=10,928). Regression analyses were utilized to determine associations between class membership and treatment receipt, as well as demographic characteristics and other comorbid conditions.

**Results:**

Five classes were identified with unique OUD symptom patterns: *Class 1: Asymptomatic* (71.6%), *Class 2: Tolerance/Time* (14.5%), *Class 3: Loss of Control/Pharmacological* (*LOC/Pharmacol*) (5.7%), *Class 4: Social Impairment* (2.6%), and *Class 5: Pervasive* (5.6%). Nearly all persons in the *LOC/Pharmacol, Social Impairment,* and *Pervasive* classes met criteria for OUD (98–100%); however, they differed in receipt of past-year treatment for substance use (28%, 28%, 49%, respectively). Age, race, education, insurance status, and criminal activity were also associated with treatment receipt.

**Conclusions:**

There were considerable differences in OUD symptom patterns and substance use treatment among individuals who misused opioids. The findings indicate a substantial unmet need for OUD treatment and point to patterns of heterogeneity within OUD that can inform development of treatment programs.

## Highlights

• Variation in OUD symptom patterns exists even among those with OUD.
• Latent class analysis identified five distinct OUD symptom patterns or classes.
• The classes had unique symptom profiles and other substance use problems.
• Treatment utilization varied across OUD symptom classes.
• Even in the class with highest symptom severity, less than 50% received treatment.

## 1 Introduction

In 2021, over 60 million people globally misused opioids (United Nations Office on Drugs and Crime, 2023). In the United States (US) over 9 million people misused opioids (prescription pain reliever and/or heroin), more than 5 million people suffered from opioid use disorder (OUD) (Substance Abuse and Mental Health Services Administration [SAMHSA], 2022), and more than 80,000 people died due to opioid related overdose (National Institute on Drug Abuse, 2023). Despite the public health impact of OUD and the availability of safe and effective treatments, less than a quarter of those with OUD receive pharmacologic treatment (Jones et al., 2023). However, little is known about how receipt of treatment varies across characteristics of those misusing prescription pain relievers and heroin, including severity and types of OUD symptoms, as well as demographic characteristics, other substance misuse/disorders, and behavioral and mental health problems.

Although the *Diagnostic and Statistical Manual of Mental Disorders* (5th ed.; [DSM-5]; APA, 2013) addresses variability of OUD severity, heterogeneity can also exist in OUD beyond quantitative levels, as those with a similar number of criteria (i.e., symptoms) could differ qualitatively regarding symptom patterns. Before the publication of DSM-5 (APA, 2013), OUD was assessed using *Diagnostic and Statistical Manual of Mental Disorders,* (4th ed.; [DSM-IV]; APA, 1994), which was divided into two distinct categories: abuse and dependence (Hasin et al., 2013). Fulfilling one abuse or three dependence criteria within the same 12 months was required for a diagnosis of opioid abuse or dependence, respectively (American Psychiatric Association, 1994, Hasin et al., 2013). Publication of the DSM-5 removed the division between abuse and dependence, added the criterion of craving, and contributed a quantitative component to assess severity based on the number of symptoms present: 2–3 (mild), 4–5 (moderate), and 6 or more (severe) (American Psychiatric Association, 2013, Hasin et al., 2013).

Not only is it important to understand patterns of OUD symptoms among those with OUD, it is also useful to understand subtypes of OUD symptoms among persons that misuse opioids but do not reach the threshold for a DSM diagnosis. Understanding symptom patterns and the characteristics of those who misuse opioids but do not meet diagnostic criteria is important to inform intervention strategies to prevent progression to a moderate or severe disorder.

Although several studies have found heterogeneity among individuals with OUD symptoms (Castaldelli-Maia et al., 2016, Ghandour et al., 2008, Tarrahi et al., 2015, Wu et al., 2011), little is known about 1) subtypes among persons that misuse opioids that vary by patterns of OUD symptoms, 2) the association of subtypes with other demographics and clinical characteristics, and 3) how these subtypes differ with respect to OUD treatment. A better understanding of OUD symptom subtypes is important for addressing unmet treatment needs. Therefore, we evaluated patterns of OUD symptoms among those who misuse opioids, examined how different OUD symptom subtypes were associated with sample characteristics (demographics, co-occurring substance use disorders [SUDs], other behavioral problems), and assessed the association of OUD symptom subtypes with treatment receipt.

## 2 Material and methods

### 2.1 Sample

Combined public use data from the US 2015–2018 National Survey on Drug Use and Health (NSDUH) were used for the analysis (SAMHSA, 2021). The NSDUH uses a complex sample design to provide estimates of drug use and related behaviors in the US population. The survey is conducted annually and draws a sample of the non-institutionalized population who are 12+ years old. “Non-institutionalized” is defined as anyone with an address, including persons within shelters, college dormitories, and migratory workers’ camps. However, persons with no fixed address (e.g., homeless), active-duty military personnel, and persons within jails, or healthcare facilities (e.g., mental health, long-term care) are excluded (Substance Abuse and Mental Health Services Administration, 2022). Informed consent is obtained from all participants and confidentiality of data is maintained by the SAMHSA and RTI International, who administer the survey. For the current study, the sample consisted of participants who reported misuse of opioids (heroin or prescription opioids) in the year before the survey, regardless of whether they were also using prescription opioids as prescribed. Misuse is defined by NSDUH as use in any way a doctor did not direct respondents to use prescription drugs, including use without a prescription of one's own; use in greater amounts, more often, or longer than told to take a drug; and use in any other way not directed by a doctor (Substance Abuse and Mental Health Services Administration, 2018). Individuals who reported only prescription opioid use as prescribed were excluded from the study sample.

### 2.2 Measures

In 2015–2018, NSDUH used DSM-IV to assess past-year SUDs. The survey assessed the presence of the 11 dependence and abuse symptoms related to heroin and prescription opioid misuse in the past year (APA, 1994). For the current analysis, a symptom was positive if it was endorsed for either heroin or prescription opioids. A proxy DSM-5 definition of OUD severity was also assessed using available data, which included all DSM-5 symptoms except craving (APA, 2013). Legal problems associated with opioids was included in the current analysis, although it was omitted in DSM-5 (American Psychiatric Association, 1994, American Psychiatric Association, 2013). Other measures included past-year SUD, number of non-opioid SUDs, depression, demographic characteristics (age, sex, race, education, insurance), criminal activity (committing a crime in the past year), and past-year treatment for illicit drug use. For those reporting past-year treatment for illicit drug use, most recent (last or current) treatment for opioids was assessed.

### 2.3 Analysis

Latent class analysis (LCA) was used to determine classes within the sample based on participants’ endorsement of the 11 DSM-IV OUD abuse and dependence symptoms in the past year. Because this analysis included a subset of NSDUH respondents who misused opioids, the study used a sample-based, rather than population-based, analysis and was conducted using unweighted survey data, including data from respondents with no missing symptom data. Fit statistics, including Akaike information criterion (AIC), Bayesian information criterion (BIC), and entropy, as well as interpretability of classes, were used to determine the number of classes that provided the best fit to the data (Weller et al., 2020). For descriptive analyses, respondents were assigned to a class using modal posterior probability of class membership. Descriptive statistics on demographics, characteristics of opioid misuse, concurrent non-opioid SUDs, and behavioral characteristics were estimated for each class.

Multiple logistic regression estimated the association of class membership, as well as other characteristics including demographics, insurance type, past-year depression, and past-year criminal activity, with treatment for problems related to illicit substance use. An initial model estimated the unadjusted odds of treatment for each class relative to the least symptomatic class. Two additional models added terms to adjust for demographics (including age, sex, race, education) and demographics plus insurance status, past-year depression, and criminal activity.

Due to the possibility of bias in regression outcomes due to assigning participants to a class based on their modal posterior probability of class membership, a sensitivity analysis was conducted (SAS software 9.4 macro LCA_Distal_BCH version 1.1.0) (Dziak et al., 2017), which considered each participant’s probability of membership in all classes rather than fixed assignment to a class when determining the likelihood of illicit substance treatment receipt. An additional sensitivity LCA analysis was carried out using NSDUH survey weights to compare to the results of weighted versus unweighted LCA models.

All analyses were run in SAS software version 9.4 (SAS Institute Inc.). Microsoft Excel Version 16.75.2 was utilized for data visualization.

## 3 Results

The sample included 10,928 participants who had endorsed past-year opioid misuse. Most reported only prescription opioid misuse (91.9%); 8.1% had used heroin (<1% heroin only, 7.2% both heroin and prescription opioids). Most participants were non-Hispanic White (62.4%), aged 18–34 years (57.8%), and, if older than 17, had attended at least some college (54.9%).

### 3.1 Latent class analysis: Five-class model

**Fig. 1:**
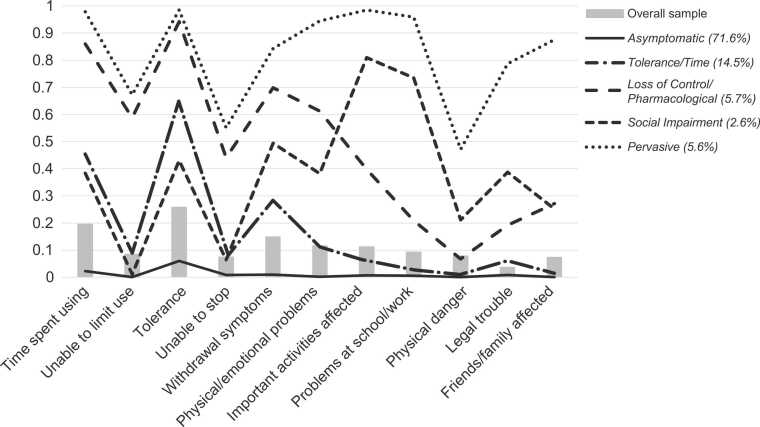
Probability of OUD symptoms by latent class membership. OUD=opioid use disorder.

Overall, the most prevalent symptoms, reported by more than 10% of the sample, were tolerance (25.8%), time spent using (19.6%), withdrawal symptoms (14.9%), physical/emotional problems (11.6%), and important activities affected (11.3%) (Fig. 1, Supplemental Table S1).

LCA results indicated that a five-class model best described the data (Table 1). Although the six-class model had a slightly lower AIC and BIC, there were minimal improvements in fit and entropy compared with the more parsimonious five-class model. Classes differed both quantitatively and qualitatively (Fig. 1, Supplemental Table S1). *Class 1: Asymptomatic* consisted of most of the sample (71.6%), and members assigned to this class had a low probability of endorsing any OUD symptoms. Participants assigned to *Class 2: Tolerance/Time* (14.5%) had moderate probabilities of spending a great deal of time using/obtaining opioids (0.45), tolerance (requiring increasing amounts of opioids to achieve the same effect; 0.65), and withdrawal (0.28). *Class 3: Loss of Control/Pharmacological* (abbreviated *LOC/Pharmacol*) (5.7%) had high probabilities of most DSM-IV dependence symptoms (time spent using, unable to limit misuse, tolerance, withdrawal, and physical/emotional problems; > 0.59), moderate probabilities of being unable to stop misuse (0.44) and having important activities affected (0.40), and a relatively low probability of abuse symptoms (< 0.27). Participants assigned to *Class 4: Social Impairment* (2.6%) had a high probability of problems maintaining work, school, or attending/performing important activities (0.73), moderate probability of other dependence symptoms and physical danger (≈0.40–0.50), and very low probability of being able to stop or limit opioid misuse (< 0.1). Participants assigned to *Class 5: Pervasive* (5.6%) had a high probability of experiencing most OUD symptoms. An LCA analysis that included sample weights yielded similar results (Supplemental Table S2).

**Table 1:** Fit statistics of class solutions of the latent class analysis.

No. of Classes	AIC	BIC	Adjusted BIC	Entropy
2	5600.79	5768.67	5695.58	0.96
3	2623.19	2878.66	2767.43	0.91
4	2140.61	2483.67	2334.31	0.92
**5**	**1669.02**	**2099.67**	**1912.17**	**0.86**
6	1574.11	2092.34	1866.71	0.86
7	1529.97	2135.79	1872.03	0.87

AIC=Akaike information criterion; BIC=Bayesian information criterion; No.=number

Bold reflects the best fitting latent class.

### 3.2 Class characteristics

#### 3.2.1 Demographics

Overall, demographics were similar between classes, with a few notable differences (Table 2). Differences by sex were small, although males were slightly overrepresented in *Pervasive*. Non-Hispanic White participants were overrepresented in *LOC/Pharmacol* and *Pervasive*, whereas minorities, including non-Hispanic Black participants and participants of Hispanic ethnicity, were more likely to be in *Social Impairment*. Compared with other age groups, adolescents (12–17 years old) were more likely to be in *Social Impairment* and less likely to be in *LOC/Pharmacol* and *Pervasive*. Individuals with a college degree were underrepresented in each of the classes associated with OUD, whereas those who completed high school were overrepresented in *LOC/Pharmacol* and *Pervasive*. There was an overrepresentation of individuals with less than a high school degree in *Social Impairment,* but underrepresentation of those with some college education.

**Table 2:** Demographic characteristics by latent class membership.

Variable	Overall Sample n 10,928	*Asymptomatic* n (%) 8351 (71.6)	*Tolerance/Time* n (%) 1088 (14.5)	*Loss of Control/ Pharmacological* n (%) 615 (5.7)	*Social Impairment* n (%) 255 (2.6)	*Pervasive* n (%) 619 (5.6)	*P* value
**Sex**							
Male	5576 (51.0)	4268 (51.1)	516 (47.4)	306 (49.8)	128 (50.2)	358 (57.8)	0.0015
**Race/ethnicity**							
White	6821 (62.4)	5113 (61.2)	648 (59.6)	454 (73.8)	137 (53.7)	469 (75.8)	<0.0001
Black	1215 (11.1)	994 (11.9)	117 (10.8)	45 (7.3)	29 (11.4)	30 (4.8)	
Hispanic	1841 (16.8)	1453 (17.4)	198 (18.2)	68 (11.1)	52 (20.4)	70 (11.3)	
Other	1051 (9.6)	791 (9.5)	125 (11.5)	48 (7.8)	37 (14.5)	50 (8.1)	
**Age, years**							
12–17	1714 (15.7)	1384 (16.6)	192 (17.6)	47 (7.6)	56 (22.0)	35 (5.7)	<0.0001
18–25	3966 (36.3)	3123 (37.4)	344 (31.6)	185 (30.1)	91 (35.7)	223 (36.0)	
26–34	2347 (21.5)	1720 (20.6)	218 (20.0)	164 (26.7)	43 (16.9)	202 (32.6)	
35–49	2053 (18.8)	1515 (18.1)	213 (19.6)	154 (25.0)	42 (16.5)	129 (20.8)	
≥50	848 (7.8)	609 (7.3)	121 (11.1)	65 (10.6)	23 (9.0)	30 (4.8)	
**Level of education**							
12–17 years old	1714 (15.7)	1384 (16.6)	192 (17.6)	47 (7.6)	56 (22.0)	35 (5.7)	<0.0001
Less than high school	1443 (13.2)	1016 (12.2)	171 (15.7)	97 (15.8)	54 (21.2)	105 (17.0)	
High school graduate	2711 (24.8)	1928 (23.1)	281 (25.8)	201 (32.7)	66 (25.9)	235 (38.0)	
Some college/ associate’s degree	3478 (31.8)	2674 (32.0)	327 (30.1)	211 (34.3)	63 (24.7)	203 (32.8)	
College graduate	1582 (14.5)	1349 (16.1)	117 (10.8)	59 (9.6)	16 (6.3)	41 (6.6)	
**Insurance**							
No insurance	1571 (14.4)	1158 (13.9)	133 (12.2)	113 (18.4)	44 (17.3)	123 (19.9)	<0.0001
Government/ government supported	3754 (34.3)	2593 (31.0)	473 (43.5)	262 (42.6)	121 (47.4)	305 (49.3)	
Private	5603 (51.3)	4600 (55.1)	482 (44.3)	240 (39.0)	90 (35.3)	191 (30.8)	

#### 3.2.2 Opioid Misuse

Prescription opioids were the predominant opioid misused in all but *Pervasive*, in which 56% used both heroin and prescription opioids and 3% used only heroin in the past year (Table 3). The prevalence of daily/near daily past month opioid misuse was highest in *LOC/Pharmacol* and *Pervasive* (22.3% and 24.4%, respectively), though the percentage reporting no past-month opioid misuse ranged from 42.3% for *Pervasive* to 75.6% for *Asymptomatic*. All individuals (100%) assigned to *LOC/Pharmacol* and *Pervasive* and nearly all (98%) assigned to *Social Impairment* met criteria for DSM-IV OUD, compared with less than half (42%) of those assigned to *Tolerance/Time*. OUD was rare in *Asymptomatic,* with only 1% meeting DSM-IV OUD criteria. Although most participants assigned to *LOC/Pharmacol, Social Impairment*, and *Pervasive* met DSM-IV OUD criteria, there were differences in the distribution of OUD severity as defined by proxy DSM-5 criteria. All participants (100%) assigned to *Pervasive* met criteria for severe OUD. In contrast, *LOC/Pharmacol* was nearly evenly split between moderate and severe OUD, whereas the distribution of severity in *Social Impairment* was shifted lower, with nearly one-third (34%) meeting criteria for mild OUD, 43% for moderate OUD, and 23% for severe OUD. The distribution of classes within each level of proxy DSM-5 OUD (none, mild, moderate, severe) was also examined (Table S3). Nearly all participants without DSM-5 OUD (99%) were in *Asymptomatic*. Of the 1063 persons meeting mild OUD criteria, most (n=977, 92%) were assigned to *Tolerance/Time*, with the remainder assigned to *Social Impairment*. Persons with moderate OUD (n=507) were split mostly between *LOC/Pharmacol* (66%) and *Social Impairment* (22%), with a small number (12%) assigned to *Tolerance/Time*. About two-thirds (65%) of the 958 respondents meeting severe DSM-5 criteria were assigned to *Pervasive*, with the remainder assigned to *LOC/Pharmacol* (29%) and *Social Impairment* (6%).

**Table 3:** Opioid use characteristics by latent class membership.

Variable	Overall Sample n 10,928	*Asymptomatic* n (%) 8351 (71.6)	*Tolerance/Time* n (%) 1088 (14.5)	*Loss of Control/ Pharmacological* n (%) 615 (5.7)	*Social Impairment* n (%) 255 (2.6)	*Pervasive* n (%) 619 (5.6)	*P* value
**Opioid misuse**							
Heroin and prescription opioids	784 (7.2)	173 (2.1)	78 (7.2)	144 (23.4)	40 (15.7)	349 (56.4)	<0.0001
Heroin use only	105 (<1.0)	51 (0.6)	17 (1.6)	9 (1.5)	7 (2.7)	21 (3.4)	
Prescription opioid use only	10,039 (91.9)	8127 (97.3)	993 (91.3)	462 (75.1)	208 (81.6)	249 (40.2)	
**Opioid misuse, days per month**							
0	7601 (69.6)	6311 (75.6)	603 (55.4)	274 (44.6)	151 (59.2)	262 (42.3)	<0.0001
1–2	1386 (12.7)	1132 (13.6)	132 (12.1)	46 (7.5)	32 (12.5)	44 (7.1)	
3–5	776 (7.1)	514 (6.2)	136 (12.5)	54 (8.8)	24 (9.4)	48 (7.8)	
6–19	659 (6.0)	283 (3.4)	129 (11.9)	104 (16.9)	29 (11.4)	114 (18.4)	
20–30	506 (4.6)	111 (1.3)	88 (8.1)	137 (22.3)	19 (7.5)	151 (24.4)	
**OUD severity**							
No OUD	8400 (76.9)	8351 (100)	49 (4.5)	0	0	0	<0.0001
Mild	1063 (9.7)	0	977 (89.8)	0	86 (33.7)	0	
Moderate	507 (4.6)	0	62 (5.7)	335 (54.5)	110 (43.1)	0	
Severe	958 (8.8)	0	0	280 (45.5)	59 (23.1)	619 (100)	
Mean No. of OUD symptoms (range)	1.3 (0, 11)	0.2 (0, 1)	2.4 (1, 4)	5.4 (4, 8)	4.2 (2, 7)	9.1 (6, 11)	<0.0001

No.=number; OUD=opioid use disorder

#### 3.2.3 Prevalence of SUDs

Prevalence of non-opioid SUDs was high across all classes (Table 4). Alcohol use disorder (AUD) had the highest prevalence, ranging from 23% in *Asymptomatic* to 42% in *Social Impairment* and *Pervasive*. Cannabis use disorder (CUD) prevalence was relatively high in this sample, ranging from 12% in *Asymptomatic* to 25% in *Social Impairment*. The prevalence of other non-opioid SUDs was highest in *Pervasive,* except for hallucinogen and inhalant use disorders, which were highest in *Social Impairment*. More than one-third of participants assigned to *Social Impairment* (38%) and *Pervasive* (37%) had multiple non-opioid SUDs compared with *Asymptomatic* (8%), *Tolerance/Time* (17%) and *LOC/Pharmacol* (23%).

**Table 4:** SUD, SUD treatment and behavioral characteristics by latent class membership.

Variable	Overall Sample n 10,928	*Asymptomatic* n (%) 8351 (71.6)	*Tolerance/Time* n (%) 1088 (14.5)	*Loss of Control/ Pharmacological* n (%) 615 (5.7)	*Social Impairment* n (%) 255 (2.6)	*Pervasive* n (%) 619 (5.6)	*P* value
**SUD**							
Opioids (heroin or prescription opioids)	2063 (18.9)	118 (1.4)	461 (42.4)	615 (100)	250 (98.0)	619 (100)	<0.0001
Alcohol	2628 (24.0)	1882 (22.5)	292 (26.8)	155 (25.2)	107 (42.0)	192 (31.0)	<0.0001
Cannabis	1441 (13.2)	991 (11.9)	154 (14.2)	100 (16.3)	65 (25.5)	131 (21.2)	<0.0001
Cocaine	456 (4.2)	185 (2.2)	57 (5.2)	53 (8.6)	27 (10.6)	134 (21.6)	<0.0001
Hallucinogens	217 (2.0)	92 (1.1)	35 (3.2)	28 (4.6)	29 (11.4)	33 (5.3)	<0.0001
Inhalants	67 (0.6)	22 (0.3)	12 (1.1)	11 (1.8)	9 (3.5)	13 (2.1)	<0.0001
Methamphetamines	437 (4.0)	186 (2.2)	47 (4.3)	55 (8.9)	31 (12.2)	118 (19.1)	<0.0001
Stimulants	341 (3.1)	147 (1.8)	44 (4.0)	45 (7.3)	26 (10.2)	79 (12.8)	<0.0001
Tranquilizers	474 (4.3)	133 (1.6)	84 (7.7)	76 (12.4)	50 (19.6)	131 (21.2)	<0.0001
Sedatives	100 (0.9)	20 (0.2)	21 (1.9)	21 (3.4)	7 (2.7)	31 (5)	<0.0001
**Number of non-opioid SUDs**							
0	6859 (62.8)	5613 (67.2)	624 (57.4)	324 (52.7)	90 (35.3)	208 (33.6)	<0.0001
1	2722 (24.9)	2034 (24.4)	283 (26.0)	151 (24.6)	69 (27.1)	185 (29.9)	
2	867 (7.9)	535 (6.4)	112 (10.3)	70 (11.4)	44 (17.3)	106 (17.1)	
≥3	480 (4.4)	169 (2.0)	69 (6.3)	70 (11.4)	52 (20.4)	120 (19.4)	
**Received treatment for illicit substance use in past year**							
Yes	957 (8.8)	314 (3.8)	98 (9)	171 (27.8)	71 (27.8)	303 (48.9)	<0.0001
**Last SUD treatment, if received**							
Opioids (heroin/prescription opioids)	690 (72.1)	129 (41.1)	75 (76.5)	150 (87.7)	49 (69.0)	287 (94.7)	<0.0001
Alcohol	466 (48.7)	235 (74.8)	46 (46.9)	54 (31.6)	33 (46.5)	98 (32.3)	<0.0001
Cannabis	320 (33.4)	158 (50.3)	31 (31.6)	36 (21.1)	26 (36.6)	69 (22.8)	<0.0001
Cocaine	193 (20.2)	50 (15.9)	17 (17.3)	27 (15.8)	16 (22.5)	83 (27.4)	<0.0001
Hallucinogens	130 (13.6)	39 (12.4)	9 (9.2)	14 (8.2)	13 (18.3)	55 (18.2)	<0.0001
Inhalants	69 (7.2)	15 (4.8)	3 (3.1)	9 (5.3)	12 (16.9)	30 (9.9)	<0.0001
Methamphetamines	176 (18.4)	61 (19.4)	17 (17.3)	12 (7.0)	18 (25.4)	68 (22.4)	<0.0001
Stimulants	95 (9.9)	20 (6.4)	6 (6.1)	11 (6.4)	14 (19.7)	44 (14.5)	<0.0001
Tranquilizer	163 (17.0)	35 (11.1)	14 (14.3)	22 (12.9)	22 (31.0)	70 (23.1)	<0.0001
Sedatives	63 (6.6)	12 (3.8)	3 (3.1)	9 (5.3)	7 (9.9)	32 (10.6)	<0.0001
**Mental health**							
MDE in the past year							
No	8307 (76.0)	6632 (79.4)	753 (69.2)	392 (63.7)	153 (60.0)	377 (60.9)	<0.0001
Yes	2438 (22.3)	1605 (19.2)	311 (28.9)	205 (33.3)	97 (38.0)	220 (35.5)	
Missing	183 (1.7)	114 (1.4)	24 (2.2)	18 (2.9)	5 (2.0)	22 (3.5)	
**Committed a crime in the past year**							
No	8590 (78.6)	6885 (82.4)	814 (74.8)	440 (71.5)	158 (62.0)	293 (47.3)	<0.0001
Yes	2251 (20.6)	1410 (16.9)	265 (24.4)	169 (27.5)	91 (35.7)	316 (51.1)	
Missing	87 (0.8)	56 (0.7)	9 (0.8)	6 (1.0)	6 (2.3)	10 (1.6)	

MDE=major depressive episode; SUD=substance use disorder

#### 3.2.4 Treatment for OUD

OUD treatment was most prevalent in *Pervasive,* in which 49% reported receiving past-year treatment for illicit substance use, and nearly all reported their most recent treatment was for problems related to opioids (95%). Although most individuals assigned to *LOC/Pharmacol* and *Social Impairment* also met criteria for OUD, they were less likely to receive treatment for illicit substance use (28% each), although most reported that their most recent treatment was for opioid problems (*Social Impairment*, 69%; *LOC/Pharmacol*, 88%). Nine percent in *Tolerance/Time* received past-year treatment for illicit substance use, predominantly for opioid problems (77%). Only 4% in *Asymptomatic* received OUD treatment, with a similar likelihood of most recent treatment for opioid, alcohol, or cannabis problems (Table 4). The unadjusted odds ratios for past-year receipt of treatment compared with *Asymptomatic* were 2.53 for *Tolerance/Time,* 9.86 for *LOC/Pharmacol*, 9.88 for *Social Impairment*, and 24.54 for *Pervasive* (Table 5).

**Table 5:** Multiple logistic regression assessing relationship between identified latent classes and past-year illicit substance treatment receipt.

Variable	n	% SUD Treatment	Unadjusted OR	Unadjusted 95% CI	Model 1 aOR	Model 1 95% CI	Model 2 aOR	Model 2 95% CI
**Class**								
*Asymptomatic*	8351	3.8	1.00					
*Tolerance/Time*	1088	9.0	2.53	2.00, 3.21	2.42	1.91, 3.07	2.06	1.61, 2.64
*Loss of Control/Pharmacological*	615	27.8	9.86	8.00, 12.15	8.82	7.11, 10.92	7.69	6.14, 9.62
*Social Impairment*	255	27.8	9.88	7.35, 13.28	9.47	7.01, 12.79	7.81	5.69, 10.71
*Pervasive*	619	48.9	24.54	20.22, 29.79	21.16	17.34, 25.82	15.03	12.14, 18.62
**Sex**								
Male	5576	9.0	1.00					
Female	5352	8.5	0.94	0.82, 1.07	1.06	0.91, 1.22	1.00	0.85, 1.17
**Age, years**								
12–17	1714	6.7	1.00					
18–25	3966	7.8	1.19	0.95, 1.49	1.22	0.92, 1.63	1.47	1.09, 1.98
26–34	2347	12.1	1.92	1.53, 2.42	1.80	1.34, 2.43	2.32	1.69, 3.20
35–49	2053	9.6	1.50	1.18, 1.90	1.48	1.09, 2.02	2.08	1.49, 2.89
≥50	848	6.1	0.92	0.65, 1.29	0.92	0.62, 1.37	1.19	0.78, 1.80
**Race**								
White	6821	10.2	1.00					
Black	1215	5.3	0.50	0.38, 0.65	0.63	0.48, 0.83	0.57	0.43, 0.77
Hispanic	1841	5.5	0.51	0.41, 0.63	0.56	0.44, 0.71	0.55	0.43, 0.70
Other	1051	9.0	0.88	0.70, 1.10	0.95	0.74, 1.21	0.92	0.71, 1.18
**Education**								
Less than high school	1443	12.3	1.00					
High school graduate	2711	11.4	0.92	0.75, 1.12	0.81	0.65, 1.01	0.85	0.68, 1.07
Some college/ associate’s degree	3478	8.4	0.65	0.53, 0.79	0.65	0.52, 0.81	0.66	0.52, 0.83
College graduate	1582	4.0	0.30	0.22, 0.40	0.36	0.260, 0.50	0.42	0.30, 0.60
**Insurance**								
No Insurance	1571	8.3	1.00					
Government/ government supported	3754	13.4	1.72	1.40, 2.10			2.13	1.68, 2.70
Private	5603	5.8	0.68	0.55, 0.84			1.23	0.96, 1.58
**Mental health**								
MDE in the past year								
No	8307	7.5	1.00					
Yes	2438	12.3	1.73	1.49, 2.00			1.13	0.95, 1.34
Missing	183							
**Committed a crime in the past year**								
No	8590	6.0	1.00					
Yes	2251	18.8	3.61	3.14, 4.14			2.40	2.03, 2.83
Missing	87							

aOR=adjusted odds ratio; CI=confidence interval; MDE=major depressive episode; OR=odds ratio; SUD=substance use disorder

Adjustment for demographic characteristics (Table 5, **Model 1**) resulted in little change in the unadjusted odds of treatment for each class relative to *Asymptomatic*. A subsequent model that added insurance, depression, and criminal activity, in addition to demographic characteristics, resulted in a modest reduction in the odds ratio estimates for each class relative to *Asymptomatic*, but the inferences remained unchanged (Table 5, **Model 2**). Participant race/ethnicity, age, education status, insurance provider, and criminal history were each associated with illicit substance treatment. Participants who were of Hispanic descent or who were non-Hispanic Black, were older than 50 years, had attended college, or had private insurance were less likely to receive treatment. Individuals who had been involved in criminal activity in the past year were more likely to receive treatment (Table 5).

Similar associations between class membership and past-year illicit substance use treatment receipt were achieved across both the assigned and probabilistic (SAS version 9.4 macro LCA_Distal_BCH version 1.1.0) latent class regression models (Dziak et al., 2017), which supported the use of modal probabilities in the logistic regression.

## 4 Discussion

Results from the LCA revealed five classes with distinct OUD symptom profiles among this US population-based sample of persons with past-year opioid misuse, ranging from subtypes reporting no symptoms to almost all symptoms. Nearly all respondents assigned to the most prevalent class (*Asymptomatic*, 71%) reported no symptoms (i.e., did not meet DSM criteria for OUD). Three of the identified classes (*LOC/Pharmacol*, *Social Impairment*, and *Pervasive*) consisted of individuals who met criteria for DSM-IV or DSM-5 OUD, and in one class (*Tolerance/Time*), nearly half met DSM-IV OUD criteria (42%) and most met DSM-5 criteria (90%). Thus, results of this study, as well as those from prior research (Castaldelli-Maia et al., 2016, Ghandour et al., 2008, Tarrahi et al., 2015, Wu et al., 2011), support the existence of multiple subtypes of OUD with respect to not only profiles of OUD symptoms but also other characteristics and likelihood of treatment. These results indicate that DSM-5 is more likely to capture heterogeneity of subtypes than DSM-IV, with different levels of DSM-5 severity generally more likely to be represented in different classes. Nonetheless, LCA identified qualitative differences between classes that extended beyond quantitative DSM-5 severity. For example, although members of *Social Impairment* and *LOC/Pharmacol* reported similar average numbers of symptoms, they had distinctly different OUD symptom profiles.

The latent class profiles in this study are similar to those identified in previous studies that used LCA, although the identification of a *Social Impairment* class was unique. Four previous LCAs examined symptom profiles of OUD symptoms, including three using earlier NSDUH data (Castaldelli-Maia et al., 2016, Ghandour et al., 2008, Tarrahi et al., 2015, Wu et al., 2011). Association with treatment receipt was considered in three of the studies (Castaldelli-Maia et al., 2016, Tarrahi et al., 2015, Wu et al., 2011). However, differences in OUD symptoms utilized (dependence vs. dependence and abuse) (Castaldelli-Maia et al., 2016, Ghandour et al., 2008, Tarrahi et al., 2015) and in opioid misuse characteristics (eg, frequency of misuse, illicit vs prescription opioids) (Castaldelli-Maia et al., 2016, Ghandour et al., 2008, Tarrahi et al., 2015, Wu et al., 2011) within study samples led to differences in numbers and characteristics of classes identified. Although the numbers of classes differed between studies, the classes identified shared similarities with many of those identified here, specifically *Asymptomatic* (Castaldelli-Maia et al., 2016, Ghandour et al., 2008, Wu et al., 2011)*, Tolerance/Time* (Ghandour et al., 2008, Tarrahi et al., 2015)*, LOC/Pharmacol* (Castaldelli-Maia et al., 2016, Ghandour et al., 2008, Tarrahi et al., 2015)*,* and *Pervasive* (Castaldelli-Maia et al., 2016, Ghandour et al., 2008, Tarrahi et al., 2015, Wu et al., 2011). The identification of *Social Impairment* has not been described previously, possibly due to the wider inclusion of those with mild OUD or less frequent misuse of heroin and prescription opioids in the current study.

In addition to differences in symptom profiles and associations with OUD, there were notable differences in the distribution of demographics and substance use characteristics across classes. As observed in previous research (Siddiqui and Urman, 2022), non-Hispanic White participants were overrepresented in *LOC/Pharmacol* and *Pervasive*. Furthermore, relative to their distribution in the sample, adolescents (12–17 years old) were underrepresented in *LOC/Pharmacol* and *Pervasive* but overrepresented in *Social Impairment*. In contrast, 26- to 34-year-old participants were overrepresented in *Pervasive*, which may indicate that the most prominent symptoms in *Social Impairment*—important activities affected and problems at school/work—could be of particular relevance to adolescents. Given that adolescents who develop OUD symptoms are early in the course of OUD, problems with engagement in important activities and school might be among the first symptoms to develop (Clayton et al., 2019). Furthermore, the overrepresentation of 26- to 34-year-old participants in *Pervasive* may reflect progression of adolescents to more severe OUD. Although it is not possible to ascertain the pattern of OUD symptom development over time in this cross-sectional study, the findings suggest that compared with other age groups, emergence of these symptoms among adolescents who misuse opioids is of concern.

The prevalence of other SUDs was high in the sample, ranging from about one-third of participants with at least one other non-opioid SUD in *Asymptomatic* to two-thirds in *Social Impairment* and *Pervasive*. The *Pervasive* class was most likely to have members with two or more non-opioid SUDs. Prevalence of AUD and CUD were several times higher in this sample of individuals compared with the general population (Table 4) (Substance Abuse and Mental Health Services Administration, 2022). There was marked variation in the patterns of comorbid SUDs among the *LOC/Pharmacol, Social Impairment*, and *Pervasive* classes, in which nearly all members met criteria for DSM-IV OUD. Participants in *Social Impairment* and *Pervasive* were more likely to have each comorbid SUD than those in *LOC/Pharmacol*; perhaps most notable is that nearly one-quarter of *Social Impairment* (25%) and *Pervasive* (22%) had past-year methamphetamine use disorder compared with 7% of *LOC/Pharmacol*. Prevalence of AUD and CUD, as well as hallucinogen and inhalant use disorders, were considerably higher in *Social Impairment* than in the other two predominantly OUD classes. These findings point to the need to adapt treatment programs to address comorbid SUDs within OUD subtypes.

Unmet treatment need for OUD is well recognized, with approximately 30% of individuals with OUD receiving recent treatment (Jones et al., 2023). Results from this study indicate that nearly half of individuals in *Pervasive* received past-year treatment for an illicit substance use problem, and nearly all reported their most recent treatment was for problems related to opioid misuse. Although similar proportions of *LOC/Pharmacol* and *Social Impairment* received SUD treatment (28% for each), members of *LOC/Pharmacol* were more likely to have received their most recent treatment for opioid problems. This is likely due to the high prevalence of other SUDs in *Social Impairment*, with a large percentage of persons in this class receiving treatment for alcohol and cannabis use problems (47% and 37%, respectively) and 25% receiving their most recent treatment for methamphetamine use. These results highlight the heterogeneity of treatment receipt within individuals with opioid-related problems. Although nearly half of individuals in *Pervasive* received treatment for opioid problems, there remains a large unmet need for treatment even in this class, as well as in classes with less *Pervasive* OUD symptom profiles. Furthermore, it is useful to consider that these cross-sectional study results reflect respondents’ status at the time of interview, and individuals in classes other than *Pervasive*, including those with subthreshold OUD symptoms, are at risk for transitioning to a more problematic class. Early detection of OUD, and other SUD symptoms could provide an opportunity for early intervention to prevent progression to more severe OUD. For example, the finding that nearly 90% of individuals in *Tolerance/Time* met DSM-5 mild OUD diagnostic criteria provides an opportunity to limit potential progression.

Higher levels of OUD symptom burden were associated with treatment receipt, consistent with prior research (Castaldelli-Maia et al., 2016, Tarrahi et al., 2015, Wu et al., 2011). *LOC/Pharmacol* and *Social Impairment* had similar odds of receiving treatment despite differences in symptom profiles, with one-third of *Social Impairment* experiencing mild OUD versus none in *LOC/Pharmacol*. This suggests that, in addition to overall symptom burden, specific symptoms that reflect interference with activities and roles which are highly prevalent within *Social Impairment,* as well as the high prevalence of comorbid SUDs in this subgroup, may lead individuals to enter treatment. The association of class membership with treatment receipt was maintained after adjustment for potential confounders, though there was a slight decrease in the magnitude of odds ratios. Other factors that were associated with SUD treatment in the final model included race/ethnicity, age, education, insurance provider (private/government), and criminal history.

Racial disparities existed, and compared with non-Hispanic White participants, participants who identified as non-Hispanic Black or Hispanic were less likely to receive treatment. This is consistent with previous research that identified racial disparities in treatment for OUD, which can exist due to fear of criminalization and stigma, racial segregation of healthcare, lack of multilingual resources, and decreased access to OUD treatment providers (Andraka-Christou, 2021, Barnett et al., 2023, Chang et al., 2022, Dunphy et al., 2022, Lagisetty et al., 2019, Siddiqui and Urman, 2022, Sledge et al., 2022, Substance Abuse and Mental Health Services Administration, 2020, Wu et al., 2016).

Eighteen- to 49-year-old participants were more likely to receive treatment for OUD than adolescents (12–17 years old), after adjustment for class membership. This age-related gap in care has been described previously (Mauro et al., 2022, Welsh et al., 2022a) and highlights the need for treatment initiation in younger populations to prevent development of OUD-related problems such as HIV infection and opioid overdose (Curtin et al., 2017, Lloyd et al., 2021, Yule et al., 2018). However, treatment initiation is only one barrier, as adolescents are also less likely to remain in treatment than are adults (Mintz et al., 2020).

Participants utilizing government insurance were more likely to receive treatment, whereas individuals with private insurance were just as likely to receive treatment as those with no insurance. Government-provided insurance is more likely to cover SUD treatment than private insurance (Mojtabai et al., 2020, U.S. Centers for Medicare & Medicaid Services, 2023). The Affordable Care Act increased enrollment in Medicaid, and state Medicaid programs have expanded coverage of SUD treatment (Mojtabai et al., 2020, Olfson et al., 2021, Sledge et al., 2022). Persons who had attended at least some college were less likely to receive treatment than those with less than a high school education, which may be confounded with increased utilization of private insurance (SHADAC, 2023).

Persons who reported criminal behavior had a higher likelihood of receiving treatment, which may be due to treatment opportunities during or after incarceration. However, in 2019, pharmacologic treatment for OUD in jails was predominantly medications for opioid withdrawal, with only 19% initiating and 24% continuing medication-assisted therapy, such as buprenorphine or methadone, during custody (Maruschak et al., 2023).

Overall, these findings illustrate the heterogeneity within individuals experiencing problems related to opioid misuse and the need for OUD symptom screening, including the potential role of measurement-based care using DSM symptomatology to optimize treatment and monitor outcomes (Marsden et al., 2019), inclusive resources and care, and access to SUD treatment coverage in a variety of settings.

### 4.1 Limitations

Limitations existed but were mitigated when possible. NSDUH sampling excludes persons with no available address, including those who are unhoused and may be more likely to experience OUD (Manhapra et al., 2021, Yamamoto et al., 2019). Therefore, it is important to consider excluded populations when generalizing results. NSDUH relies on self-report of SUD symptoms using a standardized survey instrument rather than clinical diagnosis; future studies could focus on DSM symptoms assessed by clinicians to validate findings. During the included survey years, NSDUH utilized DSM-IV SUD criteria; however, a proxy DSM-5 diagnosis was also examined. Although the DSM-IV legal criterion was included in the calculation of DSM-5 severity, this symptom was uncommon and unlikely to substantively change results. Craving, added to DSM-5, is considered by many to be a key aspect of SUD and has been associated with drug use and relapse (Hasin et al., 2012, Kakko et al., 2019, Vafaie and Kober, 2022). In 2013, the national prevalence of DSM-5 craving among past-year opioid users was approximately 8% (Compton et al., 2013), though smaller studies of patients in treatment estimate the prevalence of DSM-5 craving among past-year heroin users was approximately 66% (Hasin et al., 2012). However, the literature on the discrimination between craving and other symptoms, as well its importance in diagnosing opioid use disorder, is mixed. One study (on alcohol use disorder) suggests that craving is similar to ‘continued use despite problems’(Mewton et al., 2011, Tarrahi et al., 2015). Craving likely occurs with other OUD symptoms. In Tarrahi, et al., higher OUD symptom burden was correlated with the likelihood of experiencing craving (Tarrahi et al., 2015). Further, various tools are available to measure craving, and craving is expected to vary depending on an individual’s status in recovery vs. treatment seeking vs. currently using (Goodyear and Haass-Koffler, 2020) and, as a result, might be expected to vary across classes. However, whether the inclusion of craving would substantively affect results remains unknown but can be addressed in future studies.

Due to the nature of probabilities within an LCA, assigning class membership by modal posterior probabilities introduces possibility of class misassignment and potential bias. However, the relatively high entropy of the selected class solution and examination of the distribution of posterior probabilities within the classes indicate that the risk of misassignment was low. To further support our findings and methodology, a sensitivity analysis in which class membership was used to predict treatment utilization and was based on posterior probabilities yielded similar results as models in which class membership was fixed.

Notably, even among individuals assigned to *LOC/Pharmacol* and *Pervasive*, who all met criteria for past-year moderate/severe OUD, approximately 40% reported no past-month opioid misuse, which may reflect differential recall over the time periods (one month vs. one year) or some modulation of recent use among those individuals with the most problematic use.

Although it is necessary to use sample weights to produce population-level estimates when analyzing NSDUH, it is less clear whether to use sample weights when analyzing a subset of the NSDUH sample, as well as when using regression analysis (Bollen et al., 2016). A comparison of weighted and unweighted LCA yielded nearly identical results.

The latent class models assumed measurement invariance in which the latent class structure and meaning of constructs such as specific OUD symptoms is the same across groups. For example, among adolescents, problems at school might be more salient than other OUD symptoms, contributing their overrepresentation in the Social Impairment class. Those in SUD treatment might be more aware of DSM symptoms and more likely to endorse them than those not in treatment (Putnick and Bornstein, 2016). Any bias resulting from differential measurement has not been taken into account.

Lastly, the NSDUH is a cross-sectional study that reflects respondents’ status at a single point in time. Future longitudinal studies could examine transitions in class membership over time, including changes in class membership before and following OUD treatment receipt. In addition, the NSDUH focuses on OUD symptom among those who misuse opioids in the US. Further research is necessary to understand subtypes of OUD symptoms in populations beyond the US.

## 5 Conclusion

This study identified subtypes of OUD symptom patterns that extend beyond DSM severity among a US sample of individuals who misused opioids. Although DSM-5 severity level is useful in identifying variation in OUD, there is additional variation in symptom subtypes and other characteristics that can inform patient treatment. Additionally, there was variation in OUD treatment utilization related to specific symptom profiles and gaps in treatment still exist, as more than half of those assigned to *Pervasive* and almost three-quarters of those assigned to *LOC/Pharmacol* and *Social Impairment* had not received treatment for illicit substance use in the past year. Thus, identification of subtypes among persons who misuse opioids can inform understanding of treatment gaps as well as treatment and preventive interventions targeted to subgroup needs.

## Role of Funding

The study was supported by Indivior, Inc.

## CRediT authorship contribution statement

**Miller Emily A.:** Writing – review & editing, Writing – original draft, Visualization, Validation, Methodology, Investigation, Formal analysis, Data curation, Conceptualization. **DeVeaugh-Geiss Angela M.:** Writing – review & editing, Writing – original draft, Validation, Supervision, Project administration, Methodology, Investigation, Data curation, Conceptualization. **Chilcoat Howard D.:** Writing – review & editing, Writing – original draft, Validation, Supervision, Project administration, Methodology, Investigation, Data curation, Conceptualization.

## Declaration of Competing Interest

AMD and HDC are employees of Indivior, Inc. EAM has no conflicts of interest.
