# Supplementary material for: Opioid use disorder (OUD) and treatment for opioid problems among OUD symptom subtypes in individuals misusing opioids
Source: Drug Alcohol Depend Rep. 2024 Feb 14;10:100220. doi: 10.1016/j.dadr.2024.100220 (PMC10897812; doi:10.1016/j.dadr.2024.100220)
Supplement: Supplementary file 1 — Supplementary material [file mmc1.docx]

**Supplemental Table S1. Latent class probabilities of endorsing DSM-IV OUD symptoms**

|  | **Overall Sample (n=10,928)** | ***Asymptomatic***  **(71.6%)** | ***Tolerance/Time***  **(14.5%)** | ***Loss of Control/***  ***Pharmacological***  **(5.7%)** | ***Social Impairment*** **(2.6%)** | ***Pervasive***  **(5.6%)** |
| --- | --- | --- | --- | --- | --- | --- |
| Time spent using | 0.196 | 0.0231 | 0.4538 | 0.8598 | 0.3836 | 0.9789 |
| Unable to limit use | 0.085 | 0.0008 | 0.0889 | 0.5875 | 0.0107 | 0.6728 |
| Tolerance | 0.258 | 0.0600 | 0.6498 | 0.9420 | 0.4305 | 0.9854 |
| Unable to stop | 0.075 | 0.0085 | 0.0735 | 0.4406 | 0.0644 | 0.5509 |
| Withdrawal symptoms | 0.149 | 0.0101 | 0.2839 | 0.6988 | 0.4935 | 0.8436 |
| Physical/emotional problems | 0.116 | 0.0019 | 0.1117 | 0.6126 | 0.3816 | 0.9447 |
| Important activities affected | 0.113 | 0.0071 | 0.0620 | 0.3983 | 0.8096 | 0.9845 |
| Problems at school/work | 0.093 | 0.0061 | 0.0267 | 0.2093 | 0.7345 | 0.9584 |
| Physical danger | 0.079 | 0.0076 | 0.0610 | 0.1914 | 0.3866 | 0.7870 |
| Legal trouble | 0.037 | 0.0004 | 0.0091 | 0.0674 | 0.2118 | 0.4716 |
| Friends/family affected | 0.074 | 0.0008 | 0.0153 | 0.2709 | 0.2514 | 0.8761 |

DSM-IV: *Diagnostic and Statistical Manual of Mental Disorders*, fourth edition; OUD, opioid use disorder

**Supplemental Table S2. Weighted latent class probabilities of endorsing DSM-IV OUD symptoms**

|  | **Overall Sample (n=10,928)** | ***Asymptomatic***  **(69.0%)** | ***Tolerance/Time***  **(16.0%)** | ***Loss of Control/***  ***Pharmacological***  **(6.5%)** | ***Social Impairment***  **(3.0%)** | ***Pervasive***  **(5.5%)** |
| --- | --- | --- | --- | --- | --- | --- |
| Time spent using | 0.194 | 0.0170 | 0.3900 | 0.8235 | 0.3807 | 0.9881 |
| Unable to limit use | 0.087 | 0.0008 | 0.0707 | 0.5421 | 0.0272 | 0.6999 |
| Tolerance | 0.266 | 0.0480 | 0.6285 | 0.9428 | 0.5514 | 0.9898 |
| Unable to stop | 0.086 | 0.0088 | 0.0948 | 0.4518 | 0.0917 | 0.5853 |
| Withdrawal symptoms | 0.152 | 0.0048 | 0.2400 | 0.7358 | 0.5200 | 0.8441 |
| Physical/emotional problems | 0.122 | 0.0029 | 0.0748 | 0.6580 | 0.4745 | 0.9304 |
| Important activities affected | 0.112 | 0.0061 | 0.0527 | 0.3275 | 0.7937 | 0.9785 |
| Problems at school/work | 0.086 | 0.0049 | 0.0154 | 0.0990 | 0.6982 | 0.9496 |
| Physical danger | 0.074 | 0.0105 | 0.0420 | 0.1237 | 0.2616 | 0.7884 |
| Legal trouble | 0.033 | 0.0005 | 0.0050 | 0.0411 | 0.1486 | 0.4428 |
| Friends/family affected | 0.071 | 0.0005 | 0.0091 | 0.2042 | 0.2782 | 0.8673 |

DSM-IV=*Diagnostic and Statistical Manual of Mental Disorders*, (4th ed.); OUD=opioid use disorder

**Supplementary Table S3. Distribution of latent classes within proxy DSM-5 OUD severity levels**

| **OUD severity** | **No OUD  (<2 symptoms)**  **n (%)  (n=8,400)** | **Mild  (2–3 symptoms)**  **n (%) (n=1,063)** | **Moderate  (4–5 symptoms)**  **n (%) (n=507)** | **Severe  (≥6 symptoms)**  **n (%) (n=958)** |
| --- | --- | --- | --- | --- |
| *Asymptomatic* | 8351 (99.4%) | 0 (0.0%) | 0 (0.0%) | 0 (0.0%) |
| *Tolerance/Time* | 49 (0.6%) | 977 (91.9%) | 62 (12.2%) | 0 (0.0%) |
| *Loss of Control/Pharmacological* | 0 (0.0%) | 0 (0.0%) | 335 (66.1%) | 280 (29.2%) |
| *Social Impairment* | 0 (0.0%) | 86 (8.1%) | 110 (21.7%) | 59 (6.2%) |
| *Pervasive* | 0 (0.0%) | 0 (0.0%) | 0 (0.0%) | 619 (64.6%) |

DSM-5=*Diagnostic and Statistical Manual of Mental Disorders* (5th ed.); OUD=opioid use disorder
